# Rho GTPases in the Amygdala—A Switch for Fears?

**DOI:** 10.3390/cells9091972

**Published:** 2020-08-26

**Authors:** Tasnuva Sarowar, Andreas M. Grabrucker

**Affiliations:** 1 Cellular Neurobiology and Neuro-Nanotechnology Lab, Department of Biological Sciences, University of Limerick, V94PH61 Limerick, Ireland; t.sarowar@gmail.com; 2 Bernal Institute, University of Limerick, V94PH61 Limerick, Ireland; 3 Health Research Institute (HRI), University of Limerick, V94PH61 Limerick, Ireland

**Keywords:** Rho, GTPase, Rich2, synapse, amygdala, phobia, Rac, Cdc42, anxiety, fear memory

## Abstract

Fear is a fundamental evolutionary process for survival. However, excess or irrational fear hampers normal activity and leads to phobia. The amygdala is the primary brain region associated with fear learning and conditioning. There, Rho GTPases are molecular switches that act as signaling molecules for further downstream processes that modulate, among others, dendritic spine morphogenesis and thereby play a role in fear conditioning. The three main Rho GTPases—RhoA, Rac1, and Cdc42, together with their modulators, are known to be involved in many psychiatric disorders that affect the amygdala′s fear conditioning mechanism. Rich2, a RhoGAP mainly for Rac1 and Cdc42, has been studied extensively in such regard. Here, we will discuss these effectors, along with Rich2, as a molecular switch for fears, especially in the amygdala. Understanding the role of Rho GTPases in fear controlling could be beneficial for the development of therapeutic strategies targeting conditions with abnormal fear/anxiety-like behaviors.

## 1. Introduction

All mammals exhibit fear and anxiety as part of their evolutionary survival strategy. Fear or adaptive anxiety refers to a state with increased alertness and vigilance, enabling the individual to perform multiple defensive behaviors. However, excessively disproportionate fear with no trigger can give rise to anxiety disorders and phobia [1]. Over 28% of adults experience or exhibit some form of anxiety in their lifetime [2]. Evidence suggests that human neuropsychiatric disorders related to anxiety arise from abnormal fear learning mechanisms and unusual activity patterns in cerebral networks [3,4]. Various animal models have been developed to study anxiety and fear. In general, fear learning is well conserved among species, and studies from animal models greatly help understand the human condition [5].

Different kinds of tests have been designed to evaluate anxiety-related behaviors in rodents. These include the elevated plus maze (EPM), open field test (OF), light-dark box (LDB), and free choice exploratory (FCE) paradigm. In general, the tests are designed to assess active avoidance of potentially unsafe, dangerous, or predatory areas; like the closed arm of the EPM vs. the open arm, the center region of the OF vs. the corners, or the dark box in the LDB vs. the lit compartment [2,6]. Rodents that are more anxiety-prone tend to avoid open or brightly-lit areas in these tests, mimicking the human condition.

The amygdala is the main region for fear processing and fear memory [7]. Different subregions of the amygdala have been associated with different phobias [8]. Additionally, neuropsychiatric conditions, like autism spectrum disorders (ASD) and post-traumatic stress disorders, have been linked with abnormal amygdala activity [9,10]. For example, amygdala hypo-perfusion has been detected via positron emission tomography (PET) in Phelan McDermid syndrome, a disorder frequently associated with ASD [11].

The Rho family of small GTPases is a subfamily of Ras small GTPases. Rho GTPases are guanosine-dependent molecular switches. They are involved in numerous cellular processes ranging from cell division, differentiation, and migration to immunological and neurobiological processes [12]. Because of their ubiquitous nature and usefulness, the expression and activity of Rho GTPases are tightly regulated. Disruption in a homeostatic situation can cause many diseases like cancer, neurological disorders, and autoimmune diseases [13]. Importantly, Rho GTPases are also expressed in the amygdala. There, Rho GTPases and their effectors [14] participate in biological processes regulating fear conditioning in relation to neuropsychiatric disorders [15,16].

## 2. The Amygdala Is the Fear Center in the Brain

From the evolutionary point of view, fear of threatening stimuli is a prerequisite for survival and environmental adaptation. In addition to innate fear, learned fear is also vital for defensive behavior. In the laboratory, Pavlovian fear conditioning is widely used as a measure for fear conditioning in rodents, where animals are trained to associate a neutral sensory stimulus or conditioned stimulus (CS) with an aversive event or unconditional stimulus (US). After several rounds of training, the CS alone can generate a behavioral or autonomic response (like freezing or increased heart rate) without the US [17]. Decades of works have established the amygdala, hippocampus, and prefrontal cortex as the major brain regions associated with fear learning. However, the amygdala is the critical brain region for fear memory [1,18].

**Figure 1:**
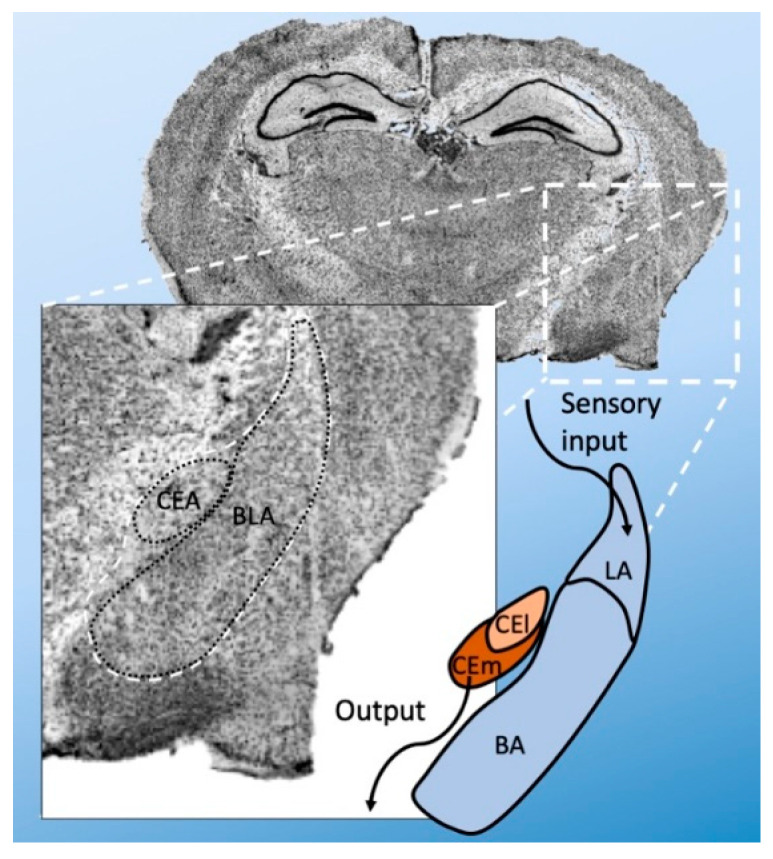
Anatomical subdivision of the amygdala (i.e., of the mouse). The amygdala is mainly subdivided into basolateral amygdala (BLA) and central nuclei (CEA). The BLA is further divided into basolateral (BA) and lateral amygdala (LA). The CEA is subdivided into a lateral (CEl) and medial (CEm) part. Broadly, the LA is the point of sensory input, and CEm is the main point of output to the brain stem.

Anatomically, the amygdala is divided into several nuclei—the basolateral (BLA) and central (CEA) nuclei [19]. The BLA is further subdivided into basolateral (BA), basomedial (BM), and lateral amygdala (LA). The CEA can be divided into a lateral (CEl) and medial (CEm) part (Figure 1). There are also intercalated cell masses (ICMs). The LA is the primary point of entry for sensory inputs, whereas the CEm is the primary source of projections to the brainstem. However, the transfer from LA to CEm is gated by other amygdala regions and their distinct cell types. The BLA receives sensory information from the thalamus and prefrontal cortex via the LA and sends signals to the CEA, where further processing occurs.

The majority of BLA neurons are spiny glutamatergic neurons (with a minority of GABAergic interneurons) [20]. CEl and CEm mainly contain GABAergic neurons. The ICMs are small cell clusters and consist of more dopamine type-1 and µ-opioid-receptor expressing cells [21]. The sensory and synaptic outputs in the amygdala heavily depend on GABAergic inhibitory outputs. They are crucial to anxiety-related fear memory, and many GABA receptor agonists are used as anxiolytic agents [22].

There are high intrinsic and extrinsic connectivities of the amygdala [23], controlling which synapses will be strengthened upon exposure to fear conditioning stimuli [24]. Many studies in the 1990s have established the LA as the principle synaptic plasticity site of Pavlovian fear [25]. Optogenetic studies have revealed pre- and postsynaptic activity for the generation of long-term potentiation (LTP) in the LA [26,27]. Accordingly, alteration of thalamic-amygdala synapses affects fear memories [28]. Intriguingly, critical molecular mechanisms of synaptic plasticity are regulated by Rho GTPases.

## 3. Rho GTPases and Their Regulation

**Figure 2:**
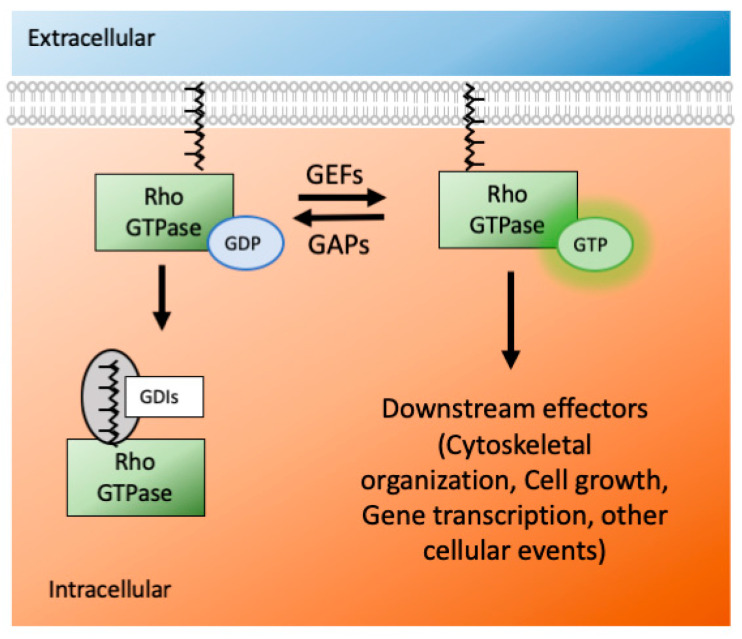
The Rho GTPases act as molecular switches in the cellular environment. They are active when they are GTP bound and inactive when they are GDP bound. GAPs and GEFs do the exchanges between GTP and GDP. When not active, the Rho GTPases remain in the cytoplasm using GDIs. Via geranylgeranylation using the enzyme GGT, they appear on the surface of the plasma membrane. (GDP-guanosine diphosphate, GTP-guanosine triphosphate, GAP-GTPase activating protein, GEF-guanosine activating factors, GDI-guanosine dissociation inhibitor, GGT-geranylgeranyl transferase).

The Ras superfamily consists of five smaller subfamilies—Ras, Rho, Ran, Rab, and Arf; based on their sequence, structure, and function [29]. The Rho subfamily has almost 20 members, and they are mostly associated with actin organization and gene expression. Rho GTPases are active when they are GTP bound and inactive when they are GDP bound. This switch from an active to inactive state (and vice versa) is modulated by many GTPase effectors, namely guanosine nucleotide exchange factors (GEFs), GTPases activating proteins (GAPs) and guanine nucleotide dissociation inhibitors (GDIs) (Figure 2). The effectors control the GTPases′ activity via several mechanisms: conformational change, translocation to membranes, and relief of auto-inhibitory intramolecular interactions [30].

Rho GTPases are activated by GEF proteins and inactivated by GAPs. A GEF binds with GDP-bound inactive Rho GTPases and forms a low-affinity complex. After nucleotide dissociation, the low-affinity complex is converted into a nucleotide-free/GEF high-affinity complex via conformational change. Upon GTP binding, the complex is converted again into a low-affinity complex, and eventually, the GEF is released [31,32]. The Rho GTPase subfamily has more than 66 GAPs, further increasing control of their activity [33]. Even though each Rho GTPase has intrinsic hydrolysis activity, the GAP proteins accelerate it by several orders of magnitude, which ensures a rapid turn-over [34]. Thus, via hydrolysis of GTP to GDP, GAPs inactivate the GTPases and prevent them from being constitutively active. For the interaction between Rho GTPases and GAPs/GEFs, the proteins have to be membrane-bound on the cell surface. The GDIs give another level of control of GTPase actions. The GDIs bind with the inactive forms of small GTPases and sequester them inside the cellular cytoplasm. They are unique to only Rho and Rab subfamily of Ras proteins [35].

The most well-known and best characterized Rho GTPase members are RhoA (Ras homologous member A), Rac1 (Ras-related C3 botulinum toxin substrate 1), and Cdc42 (cell division cycle 42). Genomic duplication of RhoA gave rise to RhoC, while RhoB is a retrotransposon of RhoA. There are also Cdc42-like (for example, TC10 and TCL) and Rac1-like (Rac2 and Rac3) proteins [36]. Each member activates some kinases or other signaling proteins that usually mediate actin dynamics. Through this, Rho GTPases have many roles in neuronal differentiation, migration, polarity establishment, morphogenesis, axon growth and guidance, and neurite outgrowth [37]. Rho GTPases affect various actin-binding and modulating proteins. For example, both Cdc42 and Rac1 can activate PAK1 (p21 activated kinase), LIMK1 (LIM kinase 1), and cofilin [38]. On the other hand, the main effector for RhoA is ROCK (Rho-associated coiled-coil containing protein kinase) [39].

## 4. Rho GTPases in Synaptic Spine Plasticity 

The tiny, actin-rich protrusions on the surface of neuronal dendrites are dendritic spines, which harbor the postsynaptic density of excitatory synapses and receive synaptic inputs [40,41,42]. Dendritic spines are dynamic and heterogeneous; depending on the synaptic strength, they can undergo size changes, a process termed spine morphogenesis [43]. There are different subtypes of the spine in terms of shape and activity. A “mature” spine usually has a short neck with a big head, mimicking a mushroom’s shape. An “immature”" spine usually has a long neck. These spines are referred to as filopodia or thin shaped [44].

**Figure 3:**
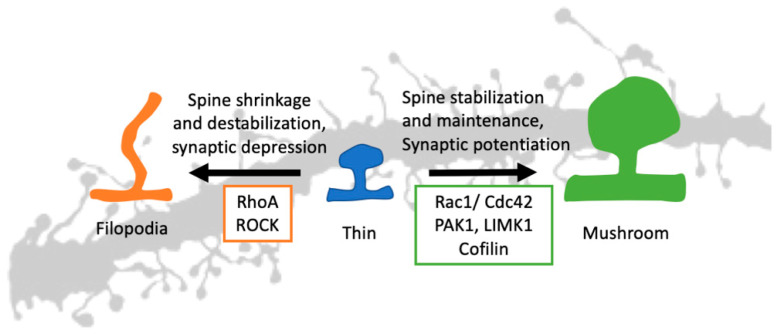
Rho GTPases in spine morphogenesis. A thin, immature spine can stabilize using synaptic potentiation and Rac1/Cdc42 signaling. Using the downstream signaling and actin effectors, both Rac1 and Cdc42 can stabilize an immature spine resulting in a mature mushroom-shaped spine. The RhoA and synaptic depression do the opposite. The RhoA signaling pathway, along with ROCK, can destabilize the spine and give it a filopodia shape. (Rac1: Ras-related C3 botulinum toxin substrate 1, Cdc42: Cell division control protein 42 homolog, RhoA: Ras homolog gene family, member A, PAK1: p21-activated kinase 1, LIMK1: LIM domain kinase 1, ROCK: Rho-associated, coiled-coil-containing protein kinase).

Due to the differences in shape and size, different spines can harbor a different set of proteins in their postsynaptic density (PSD). The structural components of the PSD include different types of metabotropic and ionotropic glutamatergic receptors (e.g., AMPA and NMDA receptors), actin effectors, transporters, ion channels, and various scaffolding proteins (like Shank, PSD95, Homer-1) [45,46]. The scaffolding proteins are the central hub in the PSD, and they bind with receptors as well as Rho GTPases. This establishes the GTPases and their effectors at the heart of spine morphogenesis [47]. They can participate in this process via their effect on actin polymerization. The effects of three main Rho GTPases on spine morphogenesis are summarized in Table 1. The downstream effectors of the main Rho GTPases and their roles in spine morphogenesis are illustrated in Figure 3.

Actin can remain in either monomeric G (globular) state or polymeric F (filamentous) state. The actin effectors change the equilibrium between these two states [53,54]. In the case of excitatory signal input or long-term potentiation (LTP), the equilibrium is shifted more towards F actin and, hence, the spine enlargement [55]. In the case of inhibitory input or long-term depression (LTP), the opposite happens, and the spine shrinks [56] (Figure 3). The change in spine size is accompanied by membrane polarization/depolarization, synaptic receptor activation/deactivation, and calcium influx/efflux [57].

Geranylgeranultransferase I (GGT) is a protein prenyltransferase that catalyzes the attachment of a prenyl lipid anchor to the carboxy-terminal of various proteins, including RhoA, Rac1 and Cdc42. It facilitates the tethering of that protein on the plasma membrane [58]. In line with the roles of the Rho GTPases, GGT promotes dendritic morphogenesis in vitro [59]. Inhibition of GGT and, thus, prevention of Rac1 activation on the cell surface decreases dendritic spine density in several brain regions [60]. 

Aberration in spine morphogenesis has been associated with neuropsychiatric disorders for a long time [61,62]. For example, an analysis of the post-mortem brain of ASD patients showed altered spine morphology [63]. Additionally, in many forms of intellectual disability, disruptions in spine morphogenesis have been reported [61]. Thus, Rho GTPases in spine morphogenesis may significantly contribute to such disorders, and altered fear/anxiety behavior in parallel.

## 5. Rho GTPases and Fear Conditioning in the Amygdala

The role of Rho GTPases in fear conditioning and consolidation is not well studied so far. However, evidence emerged for their essential role in these processes. For example, prenatal exposure to valproic acid induces anxiety-like behavior and changes the gene expression of Rho GTPases in the amygdala of mice [64]. Cat odor can induce anxiety-like behavior in rats. This reaction is embedded ethologically in their genetic memory. When male rats are exposed to cloth containing cat urine, they show anxiety-like behavior and try to avoid the cloth which suppresses their exploratory home cage activity [65]. This odor stimulus avoidance is accompanied by gene expression changes, especially the gene encoding RhoGAP 4 (Arhgap4) and RhoGEF KIAA0377.

Some of our understandings on how Rho GTPases work in the amygdala come from drug addiction and withdrawal studies. Using a conditional place aversion model, it was established that actin polymerization in the amygdala is responsible for forming aversive memory after morphine withdrawal [66]. A further study showed that the actin polymerization is mediated by the RhoA-ROCK signaling pathway and requires NMDA receptor activation [67]. The Rho-ROCK pathway in the lateral amygdala is also part of the nitric oxide driven retrograde signaling in the context of Pavlovian fear conditioning [68]. In addition, the administration of the stress hormone corticosterone on amygdaloid cell lines resulted in alteration in serotonin receptor types, and changes in RhoA and Cdc42 expression [69]. Decreased levels of Cdc42 in the forebrain (including the amygdala) lead to impairment in LTP formation and remote memory recall [52].

Additionally, Rac1 has been studied to some extent in the context of fear conditioning. Martinez et al. reported that Rac activation is increased after associative fear learning, which can be blocked using an NMDA receptor channel blocker [70]. Further, the extinction of contextual fear memory is mediated by Rac1 downregulation [71]. Before training, activation of cerebral RhoA and Rac1 enhances fear memory [72]. Rac1 inhibition using NSC23766 in the BLA after memory retrieval disrupted the reconsolidation of auditory fear memory [73]. Moreover, a mouse model was generated with conditional deletion of Rac1 in the BLA using an alpha CaMKII promoter and the stereotaxic application of AAV encoding Cre recombinase. This conditional deletion of Rac1 in excitatory neurons in the BLA impaired both short- and long-term memories [74]. Using optogenetics techniques, Das et al. have shown that activation of Rac1 in the amygdala induces PAK1 phosphorylation and inhibits long-term (but not short-term) memory formation [75]. Rac1 also has a role in the extinction of aversive memory. The knock-down of Rac1 via shRNA in the ventromedial prefrontal cortex suppressed conditioned place aversion [76]. Together, these findings indicate that Rho GTPases and some of their modulators that are expressed in amygdaloid brain regions (Table 2) are important players in fear-related processes mediated by the amygdala. 

## 6. The Critical Role of Rich2 in Fear Modulation

Rich2 (RhoGAP interacting with CIP4 homolog 2) was first identified as an interaction partner of Shank3 (SH3 and multiple ankyrin repeat domains 3), a very well-characterized gene associated with ASD [47]. Rich2 has an N-terminal BAR (Bin/Amphiphysin/Rvs) domain, RhoGAP domain, a proline-rich domain, and a C-terminal STAL motif. Yeast two-hybrid assays showed that using the STAL motif, Rich2 interacts with the PDZ domain of Shank3 [86]. Rich2 is expressed in different brain regions, including the hippocampus, cerebellum, cortex, and amygdala, and localized to excitatory synapses throughout different ages [87]. In rats, *Rich2* is located on 10q23 and codes for an 818 amino acid containing protein with 22 exons and 21 introns [88].

### 6.1. Rich2 Deletion Induces Specific Behavioral Changes in Mice

The Rich2 knock-out mice have been studied extensively using different behavioral tests [79,86]. Because of the interaction with Shank3, it was anticipated that Rich2 deletion might result in some autism features. Indeed, the Rich2 knock-out mice showed an increase in stereotypic behavior. In addition, the deletion of Rich2 impaired motor learning in knock-out animals. Most interestingly, specific impairments in the novel object recognition test were observed. When there was an object present in the open field arena, the knock-out mice significantly preferred the non-object zone over the object zone. Even when the object color or shape changes, i.e., mimics a four-legged animal, the results remained the same. Object phobia is rarely reported in animal models. To our knowledge, only mice lacking or over-expressing Neuroserpin, a serine protease inhibitor, show a similar phenotype [89].

### 6.2. Rich2 Deactivates Different Rho GTPases in Different Brain Regions

The expression and activity of Rho GTPases are tightly controlled via differential and spatial expression. A large number of GEF, GAP, and GDI proteins also help along this line. Usually, GTPase modulators interact with both Rac1 and Cdc42 [90,91]. However, RhoGEF H1 can interact with RhoA and Rac1 [92], while the GEF proteins Vav and Vav2 bind all three of the main Rho GTPases [90,93]. In a cell-free assay, the RhoGAP Rich2 exerts its GAP activity on RhoA, Rac1, and Cdc42 [94]. However, in vivo, Rich2 has been shown to activate Rac1 and Cdc42 in the hippocampus, but RhoA in the amygdala [79,86,87]. In line with this, in hippocampal cell culture, Rich2 deletion leads to over-activation of Rac1. Usually, RhoA and Rac1 have the opposite effect of spine morphogenesis [95]. Rac1 promotes spine formation, while RhoA inhibits the process [96]. Therefore, the brain-region specific activity of Rich2 is crucial to modulate spine morphogenesis in different directions in different brain regions. 

### 6.3. Spine Morphology in Rich2 Knock-Out Animals

Rich2 possesses an N-BAR domain. Its function is associated with the induction of receptor-mediated membrane curvature [97], a process required for vesicle recycling. In line with this, it has been shown that Rich2 deletion alters the PSD composition [79,86], especially for the AMPA receptor GluA4 and NMDA receptors—GluN1 and GluN2A that are regulated by vesicle trafficking. The spine morphology was also altered in the hippocampus and amygdala of the knock-out animals. In the hippocampus, the spine area was increased, and there was a shift from the mature mushroom spine to multi-lobbed spines. Electron microscopy revealed an increase in PSD density per optic field without any alteration in PSD length and width. Such alteration can be well explained in terms of the over-activation of Rac1. Further, the downstream actin effector for Rac1 mediated signaling was shown to be EPS8, an actin capping protein [98]. Therefore, the formation of F actin from G actin was significantly faster in the knock-out animals. Thus, the deletion of Rich2 over-activates Rac1 in the hippocampus, and drives the spine morphology to mature spines via recruiting EPS8.

However, in the amygdala, Rich2 deletion over-activates RhoA. Rac1 and RhoA have an antagonistic effect on spine morphogenesis. In line with this, the amygdala′s spine morphology was driven towards an immature filopodia shape, without altering the spine density. Furthermore, the equilibrium between G and F actin was shifted in the direction of G actin. Thus, Rich2 deletion affects different brain regions in different ways, directed by the Rho GTPase it acts on. 

### 6.4. Deletion of Rich2 Affects the Immediate-Early Gene Expression in the Amygdala

**Figure 4:**
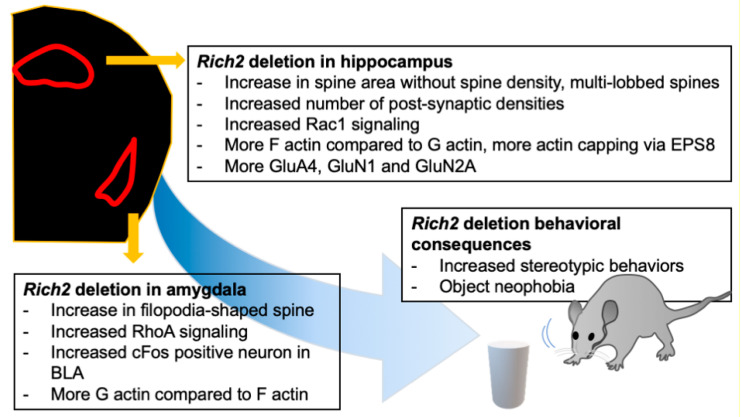
The effect of Rich2 deletion in different brain regions. Rich2 deletion affects different brain regions differently. Rich2 deletion increases stereotypic behavior and object phobia. Knock-out of Rich2 in the hippocampus shifts the spine morphology towards multi-lobbed spines, along with increased Rac1 signaling, whereas in the amygdala, the spines are shifted towards filopodia-shaped spines, along with an increase in RhoA signaling. There are also alterations in F and G actin equilibrium, immediate early gene expression, and synaptic signaling.

Activation of amygdala neurons alters the expression of immediate early genes. One of such genes is *cFos*. The cFos positive puncta in the BLA of the amygdala of the Rich2 knock-out mice were increased compared to wild-type mice [79]. RhoA can activate cFos in non-neuronal cells [99]. The same holds for neuronal cells in the amygdala as well. Thus, normally, Rich2 may keep amygdala activation in check by stabilizing excitatory synapses and reducing cFos activation in the BLA. Findings from the Rich2 knock-out animals are summarized in Figure 4.

### 6.5. Shank3-Rich2 Interaction in the Context of Fear Learning

*Shank3* is one of the most studied genes associated with ASD. Several mouse models have been generated to understand the pathophysiology of the disease, including mice with partial deletion and point mutations [100,101,102,103]. For most of the models, autism-related phenotypes were investigated extensively. Interestingly, some of the models also exhibited abnormal anxiety, fear memory, and object recognition. For example, a mouse model lacking exon 4–9 of *Shank3* exhibited object recognition deficits [104]. Another study with full *Shank3* deletion showed increased anxiety-like behavior of knock-out and heterozygous animals compared to wild-type animals [105]. Another group reported impaired contextual fear learning in the Shank3 knock-out mouse model [106]. However, to the best of our knowledge, no study has been carried out to specifically investigate the function of Shank3 in the amygdala even though *Shank3* has been reported to be expressed there [107]. 

The functional interaction between Shank3 and Rich2 has been studied extensively in vitro [94,108]. Upon LTP induction, their interaction is increased. Mutated Rich2 without Shank3 interacting motif, is not well tolerated in hippocampal culture and long-term application of this version of Rich2 on hippocampal neuronal culture was deleterious for cell health. Shank3 without PDZ domain and, thus, unable to bind Rich2, can no longer increase receptor recycling upon cLTP induction [108]. This hints at the importance of the Shank3-Rich2 interaction for LTP mediated receptor recycling. The Rich2 knock-out mouse model showed decreased Shank3 expression in the hippocampus, which was not compensated by other Shank isoforms [86]. In addition to Rich2, Shank3 has other interaction partners linking Rho GTPase and Shank3 signaling pathways. Beta-PIX, a RhoGEF for Rac1 and Cdc42, can interact with the PDZ domain of Shank3 [88]. The downstream signaling molecule for Shank3-beta PIX is PAK, which connects Shank3 to actin remodeling [51]. IRSp53 (insulin receptor tyrosin substrate kinase 3) interacts with Shank3 with its proline-rich regions and is a downstream signaling molecule of Cdc42 [90]. There are significant overlaps between Shank3 mediated structural organization of dendritic spine, and Rho GTPase influenced actin dynamics. Thus, Shank3/Rich2 interactions may be critical for Rho GTPase mediated fear regulation in the amygdala.

## 7. Rho GTPases and Fear Modulation in Neurological/Neuropsychiatric Disorders with Anxiety and Phobia as Comorbidities

Fear is a strong and ancient emotion. However, too much or too little or unspecified fear is not suitable for day to day life and can often be life-threatening [109]. The fine line between fear and phobia is essential. Since anxiety and phobia are comorbidities in many psychiatric conditions [110,111], animal models with a phobia can shed light on these comorbidities′ causal mechanisms. It is also not surprising that disorders with anxiety and phobia as comorbidities have been associated with impaired Rho GTPase signaling.

### 7.1. Rho GTPases in Mood Disorders

The breakpoint cluster region (BCR, chromosomal location 22q11) is significantly associated with bipolar disorder [112]. The BCR codes for the RhoGAP protein p21rac. In addition, Dock9, a RhoGEF for Cdc42, has also been associated with bipolar disorder [113,114]. Further, TRIO, a GEF for RhoA, Rac1, and RhoG, has been identified as a bipolar disorder-associated gene [115]. Wolfram disease is a form of bipolar disorder, and the causative gene is *Wfs1*. Aside from other brain regions, Wfs1 protein is expressed in the amygdala [81]. In addition to memory impairment and decreased social interactions, the Wfs1 knock-out animals also exhibited increased fear behavior. It was shown that the deletion of Wfs1 leads to the downregulation of Cdc42ep5, one of the targets of Cdc42. Furthermore, the conditional knock-out mouse model of RhoGAP alpha2-chimaerin with a deletion of alpha2-chimaerin from adult hippocampus using a nestin promoter, exhibited both anxiety and depression-like behavior [116]. GIMP (Gem interacting protein) is a novel RhoGAP, which has also been associated with depression [117]. 

### 7.2. Rho GTPases in Autism Spectrum Disorders

ArhGEF10, a GEF for RhoA, has been characterized in relation to ASD. ArhGEF10 is widely expressed in different brain regions. Apart from ASD-related behaviors such as social impairments, ArhGEF10 knock-out mice exhibited altered anxiety levels [118]. Another gene that has been associated with ASD, epilepsy, dyslexia, attention deficit hyperreactivity disorder (ADHD), and schizophrenia is Autism susceptibility candidate gene 2 (*Auts2*) [119,120]. Auts2 is highly expressed in the cerebral cortex, hippocampus, and cerebellum [121]. Auts2 activates the Rac1 signaling pathway and promotes neurite extension, neuronal migration, and induces lamellipodia [122]. The Auts2 heterozygous mice exhibited ASD-like behaviors but also decreased anxiety in the open field and elevated plus maze, a decrease in freezing response while showing higher response to lower nociceptor stimuli-indicating defective auditory fear conditioning and higher acoustic startle response [120].

Dock4 or dedicator of cytokinesis 4 is a Rac1 GEF located in Autism susceptibility locus 1 (Auts1) [123]. This protein is highly expressed in the hippocampus, cortex, and cerebellum [124]. Dock4 heterozygous mice showed defective social behaviors [125]. The complete knock-out mouse model manifested impaired social novelty preference, abnormality in vocalizations, and elevated anxiety [125]. Moreover, ArgGAP32, known as RICS, p250RhoGAP, or Grit, is associated with ASD [126]. ArhGAP32 can act as a GAP for all three major GTPases [127,128]. This GAP protein is abundant in the brain including in the amygdala [82]. Knock-out mice lacking all isoforms of ArhGAP32 show abnormal cued fear-learning memory, impaired motor coordination, and ASD-related behaviors such as increased repetitive behavior, and reduced behavioral flexibility [129,130].

### 7.3. Rho GTPases in Schizophrenia (SCZ)

Cdc42 was found to be upregulated in the prefrontal cortex of schizophrenic patients [131] and several other Rho effectors have been reported to be associated with schizophrenia, such as Kalirin, known as ArhGEF24, ArhGEF11, ArhGAP18, ArhGAP33, Myosin lXb, and Chimerin 2 [15,132,133]. Nevertheless, they are yet to be studied in animal models. RAPGEF6 is a known schizophrenia-associated gene and is also linked to intellectual disability. It works as a GEF protein for RAP GTPases. The deletion of RAPGEF6 reduced fear conditioning and anxiolysis and led to less neural activation in the amygdala as measured by cFOS phosphorylation [83]. There was also a reduction in spine density and primary dendrite number in the hippocampus, along with enhanced LTP in cortico-amygdala synapses.

### 7.4. Rho GTPases in Intellectual Disability (ID)

Oligophrenin 1 (*Ophn1*) is a RhoGAP protein that can inactivate all three major Rho GTPases without specificity *in vitro*. Mutations of this protein have been associated with ASD, ID, and cerebellar hypoplasia [134,135]. The structure of Ophn1 is similar to Rich2 to some extent. It has a BAR domain, a PH domain, GAP domain, and proline-rich C-terminus [136]. This RhoGAP is expressed almost ubiquitously in the brain [84]. Deletion of *Ophn1* leads to a range of behavioral abnormalities like hyperreactivity, altered spatial memory, impaired object recognition memory, and cognitive impairment, but also a deficit in fear memory extinction [137,138,139]. The cAMP/PKA signaling pathway was found to be affected in the hippocampus and amygdala of *Ophn1* knock-out mice [139]. The knock-down of *Ophn1* leads to overexpression of RhoA and a decrease in spine density [84]. Similar to the interaction between Rich2 and Shank3 [47], Ophn1 binds the scaffolding protein Homer, and this interaction is vital for mGluR mediated signaling.

The ArhGEF9, alternatively known as collybistin, works for Cdc42 [140]; and is associated with ID, epilepsy, anxiety, and aggression [141]. Collybistin knock-out mice show reduced exploratory behavior and enhanced anxiety [142]. Collybistin is expressed in the BLA, and its deletion leads to loss of inhibitory gephyrin and GABA clusters [126,140]. Moreover, ArhGAP14 (MEGAP or SrGAP3) is a member of the Slit-Robo GTPase-activating protein subfamily and has GAP activity for Rac1 [143]. SrGAP3 is associated with ID [85,142] and ASD. It has a role in spine development and is highly expressed in several brain regions, including the amygdala [85]. Loss of SrGAP3 disrupts LTP, impairing learning and memory [143]. Genetic depletion of SrGAP3 leads to an increased basal level of Rac1 and longer dendritic spines [144]. The behavioral analysis further revealed memory problems, impaired social behavior, tics, but also altered anxiety-related behaviors, such as increased freezing time in the fear conditioning test [144].

## 8. Rho GTPases as a Potential Drug Target 

A number of Rho GTPases and their regulators have a functional role in the amygdala (Table 3).

Thus far, the first line of treatment for many neuropsychiatric disorders with anxiety and phobias as comorbidity, or anxiety disorders are GABA receptor antagonists like benzodiazepines [148]. However, that comes with a long list of side effects. The Rho GTPases are the downstream target of some antipsychotic drugs already. For example, the drug GLYX-13, an NMDA modulator, activates Rac1 (but not RhoA) to exert its anti-depressive effect [149]. Rho GTPase-related pathways are also targeted in the oligodendrocytes and neurons by the antidepressant drug Agomelatine [150]. Paeonol ameliorates the hippocampal neuronal morphology and depression in chronic unpredictable mild stress rat model via compensating the effect of upregulated Rac1/RhoA [151].

In many mouse models, overexpression of Rho GTPases that were inactive due to mutation rescued the phenotype. For example, some of the phenotypes observed after the deletion of *Dock4*, i.e., reduced spine density in the hippocampus and defective social preference, was restored by overexpression of Rac1 [125]. Over-activation of RhoA was observed in *Ophn1* knock-out animals [152]. Chronic treatment with Fasudil (a clinically-approved ROCK inhibitor) restored fear memory extinction, locomotor activity, object recognition memory, and spine morphology alterations [152].

However, there are many obstacles for targeting the small GTPases and their effectors for drug discovery. Therefore, most Ras proteins are often considered “undruggable”. The Rho GTPases are mainly involved in protein-protein interaction (PPI), and targeting PPI is always an enormous challenge. There are several ways to target the activity of Rho GTPases. One strategy could be farnesyltransferase inhibitors and geranylgeranyltransferase inhibitors that inhibit GTPases from appearing on the cell surface. Inhibition of protein prenylation is an attractive goal for reducing the overactivation of Rho GTPases [153]. An alternative approach could be targeting the downstream effectors, although they often lack specificity and develop complex feedback mechanisms [154]. GEFs are easier targets than GAPs or GDIs [12]. Examples include targeting either GTPases or GEF and inhibiting GTPase-GEF complex formation and binding to GTPases to mimic GAP, preventing GEF activation. Numerous small molecules have been studied to achieve these goals, but none has been proven to work flawlessly. Nonetheless, this drug discovery field is still attractive to the pharmaceutical industry. Especially the role of Rich2 in specific phobia-like behaviors without a generally increased anxiety could make it an attractive drug target for specific phobias.

## 9. Conclusions

Impairment in fear conditioning is not just comorbidity in neurological disorders; specific phobias may affect an individual′s day-to-day life. Therefore, understanding the process and the effect of Rho GTPases are essential to design therapeutic strategies. Animal studies have revealed several links between Rho GTPases and anxiety behavior, whether standing alone or in the context of other neurological/neuropsychiatric disorders. The fact that deletion/mutation in several Rho GTPases, GAPs and GEFs result behavior characteristic for neuropsychiatric disorders but also altered anxiety/fear responses, or both together, points at the important role of GTPase signaling in the amygdala. How exactly Rho GTPases regulate fear on molecular level remains to be determined and may be complex, as illustrated by the dual role of Rich2 that interacts with Rac1 and Cdc42 in the hippocampus, but with RhoA in the amygdala, thereby, at the same time, driving spine morphology in opposite directions in different brain regions.

## Figures and Tables

**Table 1:** Role of Rho GTPases on spine morphogenesis in vivo and in vitro.

GTPase	Study Model	Study Findings	Ref.
RhoA	Neuronal culture	Inhibits dendritic growth and dynamics; constitutive overexpression leads to spine loss	[48]
Rac1	Neuronal culture	Promotes dendritic growth and dynamics	[48]
	Hippocampal slice culture	Overexpression of dominant-negative Rac1 reduces spine density	[49]
	Conditional knock-out (alpha CaMKII promoter, deletion in the postnatal hippocampus)	Reduction in Rac1 activity and spine density, impairment in learning and memory (using Rac1 inhibitors)	[50,51]
Cdc42	Neuronal culture	Promotes dendritic growth and dynamics	[48]
	Conditional knock-out (alpha CaMKII promoter, deletion in postnatal forebrain)	Spine density of hippocampal CA1 reduced, remote memory recall was impaired	[52]

**Table 2:** Expression of Rho GTPases and their modulators in amygdala subregions in rodents.

GTPase	BLA Expression	CEA Expression	Ref.
RhoA	+ (in all parts of the amygdala)		[67]
Rac1	+	not reported	[74,77]
Cdc42	+ *		-
Kalirin	+	CEl and CEm	[78]
Rich2	+		[79]
BCR	+ *		[80]
Dock9	+ *		[80]
Trio	+ *		[80]
Wfs1	+		[81]
Auts2	+ *		[80]
Dock4	+ *		[80]
RICS	+		[82]
RAPGEF6	+		[83]
Oligophrenin 1	ubiquitous (whole brain)		[84]
MEGAP	+		[85]

* expressed in humans.

**Table 3:** The known functions of Rho GTPases and their regulators in the amygdala.

GTPase/Regulator	Function in Amygdala	Association with Disease	Ref.
Rac1	Extinction of contextual fear		[71]
	Reconsolidation of auditory fear memory		[73]
	Post-training activation is required for both long-term and short-term auditory fear memory.		[74]
	Photoactivation of Rac1 in LA impairs fear memory.		[75]
Alpha2- chimaerin (RhoGAP)	Abnormal contextual fear learning		[145]
ArhGAP4	Odor stimulus avoidance		[65]
MEGAP (ArhGAP14)	Loss of MEGAP disrupts learning and memory	ASD and ID	[143]
ArhGAP32	Knock-out showed abnormal fear learning memory and ASD-like behaviors	ASD	[129,130]
Oligophrenin 1 (RhoGAP)	Deletion leads to a deficit in fear memory extinction and alteration in the cAMP/PKA pathway	ASD, ID, and Cerebellar Hypoplasia	[134,135,139]
RhoGEF KIAA0377	Odor stimulus avoidance		[65]
Wfs1	Modulation of anxiety and fear	Bipolar disease (Wolfram disease)	[146]
TRIO (RhoGEF)	Neural Development	Bipolar disease	[115,147]
Collybistin (ArhGEF9)	Deletion leads to loss of inhibitory neurons	ID, Anxiety, Epilepsy	[131]
ArhGEF10	Knock-out increased norepinephrine and serotonin levels in the amygdala	ASD	[118]
RAPGEF6	Deletion leads to reduced fear learning and less neuronal activation	SCZ	[83]
